# Direct comparison of echocardiography speckle tracking and cardiac magnetic resonance feature tracking for quantification of right ventricular strain: a prospective intermodality study in functional mitral regurgitation

**DOI:** 10.1186/s44156-022-00011-8

**Published:** 2022-11-01

**Authors:** Justin Johannesen, Rena Fukuda, David T. Zhang, Katherine Tak, Rachel Meier, Hannah Agoglia, Evelyn Horn, Richard B. Devereux, Jonathan W. Weinsaft, Jiwon Kim

**Affiliations:** Division of Cardiology, Department of Medicine, Weill Cornell Medicine/New York Presbyterian Hospital, 525 East 68th Street, New York, NY 10021 USA

**Keywords:** Right ventricular strain, Global longitudinal strain, Speckle tracking, Feature tracking, Cardiac magnetic resonance

## Abstract

**Background:**

Functional mitral regurgitation (FMR) is a known risk factor for right ventricular dysfunction (RV_DYS_). RV global longitudinal strain (GLS) is an emerging index of RV function; however, the magnitude of agreement between RV GLS by echocardiography (echo) and cardiac magnetic resonance (CMR) and the relative utility of each modality for both the diagnosis of RV_DYS_ and prognostication of all-cause mortality and heart failure hospitalization remain unknown.

**Results:**

32% of patients had RV_DYS_ (EF < 50%) on CMR, among whom there was more advanced NYHA class and lower LV and RV ejection fraction (all p < 0.05). RV GLS was impaired in patients with RV_DYS_ whether quantified via STE or FT-CMR, with strong correlation between modalities (r = 0.81). Both STE and FT-CMR derived GLS yielded excellent detection of RV_DYS_ (AUC 0.94 for both), paralleling similar performance for free wall strain by both modalities (FT-CMR AUC 0.94, STE AUC 0.92) with lower accuracy demonstrated by STE derived septal strain (STE AUC 0.78 and FT-CMR AUC 0.92). RV S’ and TAPSE showed lower diagnostic accuracy (RV S’ AUC 0.77 and TAPSE AUC 0.81). During median follow up of 51 months (IQR 42, 60 months), all-cause mortality or HF hospitalization occurred in 25% (n = 25). Both STE and FT-CMR derived RV GLS stratified risk for adverse prognosis (STE p = 0.007, FT-CMR p = 0.005) whereas conventional RV indices, TAPSE and RV S’, did not (TAPSE p = 0.30, S’ p = 0.69).

**Conclusion:**

RV GLS is a robust marker of RV_DYS_ irrespective of modality which provides incremental diagnostic value and improves risk stratification for event free survival beyond conventional RV indices.

## Background

Functional mitral regurgitation is an established risk factor for Right Ventricular dysfunction (RV_DYS)_ due to multiple mechanisms. Mitral regurgitation can result in RV impairment due to increase in atrial volume, resulting in progressive increase in atrial pressure and increased RV afterload and end-diastolic pressure [1–3]. Additionally, relevant in functional/ischemic mitral regurgitation, left ventricular (LV) ischemia has been shown to directly impair RV contractility due to effects on RV perfusion (in LV and RV regions sharing common coronary arterial supply) [4, 5]. These factors have contributed to the growing attention on RV function among patients with functional mitral regurgitation. While echocardiography (echo) has been widely used to assess cardiac structure and function in coronary artery disease (CAD) and mitral regurgitation, two-dimensional (2D) echo assessment of the RV can often be limited due to its irregular shape, retrosternal position, and prominent trabeculations [6]. Traditional 2D markers have been shown to yield lower reproducibility compared to volumetric RV imaging [7, 8]. These limitations are particularly relevant to patients with CAD, in whom dysfunction can be regional and not reflected by one measurement in a single plane. Right ventricular global longitudinal strain (RV GLS) is an increasingly utilized measure of RV function, with the advantage of being both angle-independent and less load-dependent, that has the potential to provide more accurate RV assessment.

At present, there are two primary methodologies for RV GLS quantification. Speckle tracking echocardiography (STE) is a technique which relies on stable acoustic markers that result from positive interference of the myocardial tissue with the ultrasound beam. The position of these speckles in relation to each other can be used as a surrogate for myocardial contraction. Feature tracking cardiac magnetic resonance (FT-CMR) imaging requires high spatial resolution cardiac MRI cine images to track myocardial motion during the cardiac cycle. While RV GLS has been shown to be feasible with each modality and to provide prognostic utility [9, 10], the relative diagnostic performance of STE versus FT-CMR as well as inter-modality interchangeability of GLS is unknown.

Given the importance of early detection of RV_DYS_ in functional mitral regurgitation, this study sought to assess the relative prognostic and diagnostic value of FT-CMR and STE for RV strain in a prospective cohort of patients with functional mitral regurgitation undergoing same day CMR and transthoracic echo using a standardized protocol tailored for assessment of the RV. Study aims were three-fold: 1. To test the relative diagnostic performance of STE and FT-CMR for both global and regional RV function by the reference standard of volumetric quantification on CMR; 2. Compare RV strain based analyses by both CMR and echo to conventional RV linear performance indices; 3. Evaluate the relative prognostic utility of conventional, strain and volumetric methods for prediction of all-cause mortality or heart failure [11] hospitalization.

## Materials and methods

### Study population

The study population comprised a prospectively enrolled cohort of patients in a multimodality imaging protocol examining the right heart in functional mitral regurgitation. In all patients, comprehensive demographic data were prospectively obtained via a standardized questionnaire and review of medical history. Eligible patients had functional mitral regurgitation (≥ mild) on echo and were being considered for invasive angiography based on suspected/known obstructive CAD or prior abnormal stress test. Patients were excluded enrollment if there were contraindication to CMR or gadolinium (e.g. known/suspected hypersensitivity, GFR < 30 ml/min/1.73m^2^, intrinsic mitral valve disease (prolapse, rheumatic heart disease) or mitral valve replacement, or unrelated clinical condition (e.g. stage IV neoplasm) with life expectancy < 12 months prohibiting follow-up. Comprehensive demographic data were collected using standardized questionnaires, including cardiac risk factors and medications and mortality was recorded via follow up phone call and chart review in accordance with study protocol. All patients provided informed written consent at the time of imaging and the study was conducted with approval of the Weill Cornell Medical College (WCMC) institutional review board.

## Image acquisition and analytic protocol

### Echocardiography

Transthoracic echocardiography was performed using commercial equipment (Philips iE33 and EPIQ7 [Andover, MA]). Images were acquired in accordance with consensus guidelines, inclusive of functional and flow-based Doppler imaging. Examinations were interpreted by investigators within a high-volume laboratory for whom reproducibility has previously been documented (JK, RBD, JWW) [8, 12]. RV assessment was performed in an RV focused apical view; images were acquired at a frame rate of 60–90 Hz and RV global longitudinal strain (RV GLS) as well as septal and free wall strains were quantified. For strain analysis, the right ventricular free wall was defined as the myocardium adjacent to the lateral tricuspid annulus to the cardiac apex. The right ventricular septum was delineated from the myocardial adjacent to the medial tricuspid annulus to the cardiac apex. Strain was derived using commercial software (TomTEC [Munich, Germany]), for which automated border detection was manually adjusted to ensure optimal tracking throughout the cardiac cycle. RV 3D volumes were quantified in a full volume RV focused view; data were acquired over 4 cardiac cycles and processed using above noted dedicated software: 3D RV end-diastolic and end-systolic datasets included chamber volumes inclusive of the RV inflow and outflow tracts as respectively bordered by the tricuspid and pulmonic annuli. Quantitative analyses were also performed to obtain additional indices pertinent to RV function including RV S’ and TAPSE. RV fractional area change (FAC) was acquired by delineation of RV chamber area in end-diastole and end-systole and calculated as percentage change. Pulmonary arterial systolic pressure was obtained using the peak velocity of tricuspid regurgitant flow using the modified Bernoulli equation and right atrial pressure corresponding to inferior vena cava collapsibility. Mitral regurgitation and tricuspid regurgitation were quantified using standard American Society of Echocardiography criteria [13] and graded based on aggregate 4-point scale (1 = mild to 4 = severe). Echo parameters were quantified blinded to CMR results.

### Cardiac magnetic resonance

CMR was performed using 3.0 Tesla scanners (General Electric [Waukesha, WI]). Cardiac chamber volumes were assessed via cine-CMR (steady state free precession), which included long axis (2, 3, 4) as well as contiguous short axis slices acquired from the atrio-ventricular (tricuspid and mitral valve) annuli through the LV and RV apices that were quantified at end-diastole and end-systole for calculation of LV and RV ejection fraction (EF). RV strain was measured using cine-CMR 4-chamber view datasets for which automated border detection was performed using a commercially available program (Precession, Heart Imaging Technologies, Durham, North Carolina). Borders were then manually edited to optimize myocardial tracking to then acquire RV global (RV GLS), septal and free wall strains. Standardized clinical protocols (dedicated shimming, frequency scout pre-scans, etc.) were implemented to minimize field inhomogeneity and reduce artifacts, including banding and pulsation artifacts.

### Data analysis

Independent samples *t*-test (for continuous parametric data) and Pearson’s chi-square analysis (for categorical data) were conducted to determine the significant difference in clinical and imaging characteristics between patients with RV_DYS_ (RVEF < 50%) [14, 15] and those without. Bivariate correlations of FT-CMR and STE peak strain values were analyzed using linear regression and generation of Pearson correlation coefficients [16]. Bland Altman plots were employed to analyze agreement between FT-CMR and STE. Diagnostic performances of FT-CMR and STE RV longitudinal strain were assessed using receiver operating characteristic [2] curves. Kaplan–Meier analysis was utilized to assess the survival function stratified by impaired RV GLS on echo and CMR using threshold of RV GLS < 10%. Univariate and multivariate cox regression models were performed to determine predictors of mortality and HF hospitalization. All statistical analyses and figure generations were performed in SPSS 27.0 (SPSS Inc. [Chicago, IL]). Two-tailed significance was defined as p < 0.05.

## Results

### Population characteristics

The population comprised 100 prospectively enrolled patients with functional mitral regurgitation who underwent same day echo and CMR. 32% (n = 32) had RV_DYS_ defined as RVEF < 50%. Table 1 details clinical characteristics of the population stratified by RV_DYS…_ As shown, patients with RV_DYS_ were more likely to report symptoms of dyspnea, have advanced NYHA class and require loop diuretics (all p < 0.05). Regarding imaging parameters, as shown in Table 2, RV_DYS_ was associated with greater LV and RV chamber remodeling as evidenced by increased chamber size whether assessed via echo or CMR (p < 0.05 for all).

**Table 1:** Baseline clinical characteristics

Clinical Characteristics	Overall (n = 100)	RV_dys_ + (n = 32)*	RV_dys_- (n = 68)	p
Age (years)	67.8 ± 10.0	69.9 ± 10.1	66.8 ± 9.9	0.16
Male	80% (80)	87.5% (28)	76.5% (52)	0.20
Body surface area	1.9 ± 0.2	1.9 ± 0.2	2.0 ± 0.2	0.69
Heart rate	66.8 ± 14.6	69.9 ± 14.5	65.4 ± 14.6	0.18
Systolic blood pressure	121.9 ± 17.0	123.5 ± 12.5	121.2 ± 18.7	0.53
Diastolic blood pressure	68.8 ± 10.4	68.3 ± 10.4	69.0 ± 10.5	0.78
*CV risk factors*				
Hypertension	79% (79)	68.8% (22)	85.1% (57)	0.06
Hypercholesterolemia	76% (76)	68.8% (22)	80.6% (54)	0.20
Diabetes mellitus	50% (50)	62.5% (20)	44.8% (30)	0.10
Tobacco use	61% (61)	65.6% (21)	59.7% (40)	0.57
Family history CAD	24% (24)	21.9% (7)	25.4% (17)	0.70
Prior myocardial infarction	54% (54)	59.4% (19)	52.2% (35)	0.51
*Cardiovascular medications*				
Loop diuretic	42% (42)	62.5% (20)	32.8% (22)	**0.005**
ACEI/ARB	60% (60)	75.0% (24)	53.7% (36)	**0.04**
Aspirin	87% (87)	87.5% (28)	88.1% (59)	0.94
Thienopyridine	40% (40)	46.9% (15)	37.3% (25)	0.37
Beta-blocker	78% (78)	81.3% (26)	77.6% (52)	0.68
*Cardiovascular symptoms*				
NYHA Class (1/2/3/4)	55% (55) /24% (24) /11% (11) /6% (6)	30.0% (9) /36.7% (11)/ 16.7% (5) /16.7% (5)	69.7% (46) /19.7% (13)/ 9.1% (6) /1.5% (1)	** < 0.001**
Dyspnea	59% (59)	82.8% (24)	59.3% (35)	**0.03**
Angina	49% (49)	62.1% (18)	52.5% (31)	0.40
*Prior coronary revascularization*				
PCI	57% (57)	62.5% (20)	55.2% (37)	0.49
CABG	32% (32)	34.4% (11)	31.3% (21)	0.76
PCI or CABG	77% (77)	81.3% (26)	76.1% (51)	0.57

Bold values identify a significance of *p* < 0.05

**Table 2:** Population imaging characteristics

Imaging characteristics	Overall (n = 100)	RV_dys_+ (n = 32)*	RV_dys_- (n = 68)	p
***Echocardiography***				
*Right Ventricle*				
3D Ejection fraction (%)	52.0 ± 10.1	41.8 ± 9.3	57.0 ± 5.9	**< 0.001**
End-diastolic volume (ml)	134.6 ± 42.0	154.4 ± 41.7	125.1 ± 39.1	**0.002**
End-systolic volume (ml)	66.2 ± 31.1	90.2 ± 34.1	54.6 ± 21.8	**< 0.001**
Stroke volume (ml)	67.9 ± 18.5	61.6 ± 13.3	70.8 ± 19.9	**0.02**
S' (cm/s)	11.4 ± 3.0	9.8 ± 3.0	12.1 ± 2.7	**< 0.001**
TAPSE (cm)	1.8 ± 0.4	1.5 ± 0.3	1.9 ± 0.4	**< 0.001**
FAC (%)	35.7 ± 10.6	29.4 ± 9.8	39.1 ± 9.5	**< 0.001**
*Left Ventricle*				
Ejection fraction (%)	41.9 ± 14.6	30.6 ± 11.3	47.4 ± 12.9	** < 0.001**
LVIDd (cm)	5.9 ± 0.6	6.2 ± 0.6	5.7 ± 0.6	** < 0.001**
LVIDs (cm)	4.6 ± 0.9	5.3 ± 0.7	4.3 ± 0.8	** < 0.001**
End-diastolic volume (ml)	171.0 ± 39.0	189.2 ± 35.5	163.8 ± 38.2	**0.005**
End-systolic volume (ml)	101.5 ± 45.3	138.0 ± 42.4	86.7 ± 37.7	** < 0.001**
Stroke volume (ml)	70.5 ± 21.5	55.8 ± 19.1	76.1 ± 19.7	** < 0.001**
Myocardial mass (g)	208.7 ± 70.0	227.8 ± 70.2	199.9 ± 68.7	0.10
*Hemodynamics*				
PASP (mmHg)	37.8 ± 14.8	34.4 ± 13.4	43.9 ± 15.3	**0.004**
***Valvular Indices***				
*Mitral Regurgitation*				
Severity grade (1–4)	59% (59) 25% (25) /8% (8)/ 6% (6)	34.4% (11)/ 34.4% (11)/18.8% (6) /12.5% (4)	72.7% (48)/21.2% (14)/3.0% (2) /3.0% (2)	** < 0.001**
Regurgitant fraction (%)	37.97 ± 15.19	42.49 ± 14.72	34.70 ± 14.85	**0.04**
Regurgitant volume (ml)	38.3 ± 26.99	46.2 ± 6.4	34.3 ± 3.9	0.10
Vena contracta (cm)	0.33 ± 0.11	0.38 ± 0.11	0.29 ± 0.11	**0.002**
*Tricuspid Regurgitation*				
Severity grade (1–4)	64% (64)/ 23% (23)/ 12% (12)/ 1% (1)	47% (15)/ 28% (9)/ 25% (8)/ 0% (0)	72% (49)/ 21% (14)/ 6% (4)/ 1% (1)	**0.02**
*Peak Longitudinal Strain*				
RV global (%)	18.3 ± 5.4	12.5 ± 4.1	21.0 ± 3.5	** < 0.001**
RV free wall (%)	18.0 ± 6.7	11.6 ± 5.5	21.6 ± 4.3	** < 0.001**
RV septal (%)	11.0 ± 6.9	7.2 ± 5.7	13.2 ± 6.7	** < 0.001**
***CMR***				
*Right Ventricle*				
Ejection fraction (%)	52.0 ± 11.7	39.2 ± 10.1	58.0 ± 6.3	** < 0.001**
End-diastolic length (cm)	7.4 ± 1.2	7.9 ± 1.1	7.2 ± 1.2	**0.01**
End-diastolic width (cm)	4.2 ± 0.7	4.4 ± 0.7	4.1 ± 0.7	**0.04**
End-diastolic volume (ml)	153.0 ± 52.4	174.9 ± 58.4	142.5 ± 46.2	**0.003**
End-systolic volume (ml)	76.8 ± 42.4	109.7 ± 51.0	61.1 ± 25.8	** < 0.001**
Stroke volume (ml)	76.0 ± 22.0	65.2 ± 14.8	81.2 ± 23.0	** < 0.001**
RV myocardial mass	29.0 ± 10.2	31.7 ± 8.2	27.7 ± 10.8	0.07
Indexed (g/m^2^)	15.0 ± 4.9	16.7 ± 4.9	14.1 ± 4.7	**0.01**
*Left Ventricle*				
Ejection fraction (%)	43.2 ± 15.4	30.9 ± 10.4	48.9 ± 13.9	** < 0.001**
End-diastolic volume (ml)	200.4 ± 63.8	232.6 ± 60.6	185.2 ± 59.9	** < 0.001**
End-systolic volume (ml)	120.8 ± 62.6	163.9 ± 55.5	99.9 ± 55.0	** < 0.001**
Stroke volume (ml)	80.0 ± 23.8	68.8 ± 20.2	85.3 ± 23.6	** < 0.001**
LV myocardial mass	157.4 ± 47.0	171.2 ± 38.5	151.0 ± 49.4	**0.04**
Indexed (g/m^2^)	81.1 ± 20.8	89.1 ± 19.0	77.3 ± 20.6	**0.007**
End-diastolic diameter (cm)	6.0 ± 0.7	6.3 ± 0.6	5.8 ± 0.7	**0.002**
*Longitudinal Strain*				
RV global (%)	20.5 ± 6.8	13.7 ± 3.9	23.7 ± 5.4	** < 0.001**
RV free wall (%)	15.8 ± 7.1	9.0 ± 4.9	19.1 ± 5.5	** < 0.001**
RV septal (%)	23.9 ± 7.5	17.2 ± 5.1	27.1 ± 6.2	** < 0.001**

Bold values identify a significance of *p* < 0.05

^*^RV ejection fraction < 50%

### STE and FT-CMR RV strain

**Fig. 1:**
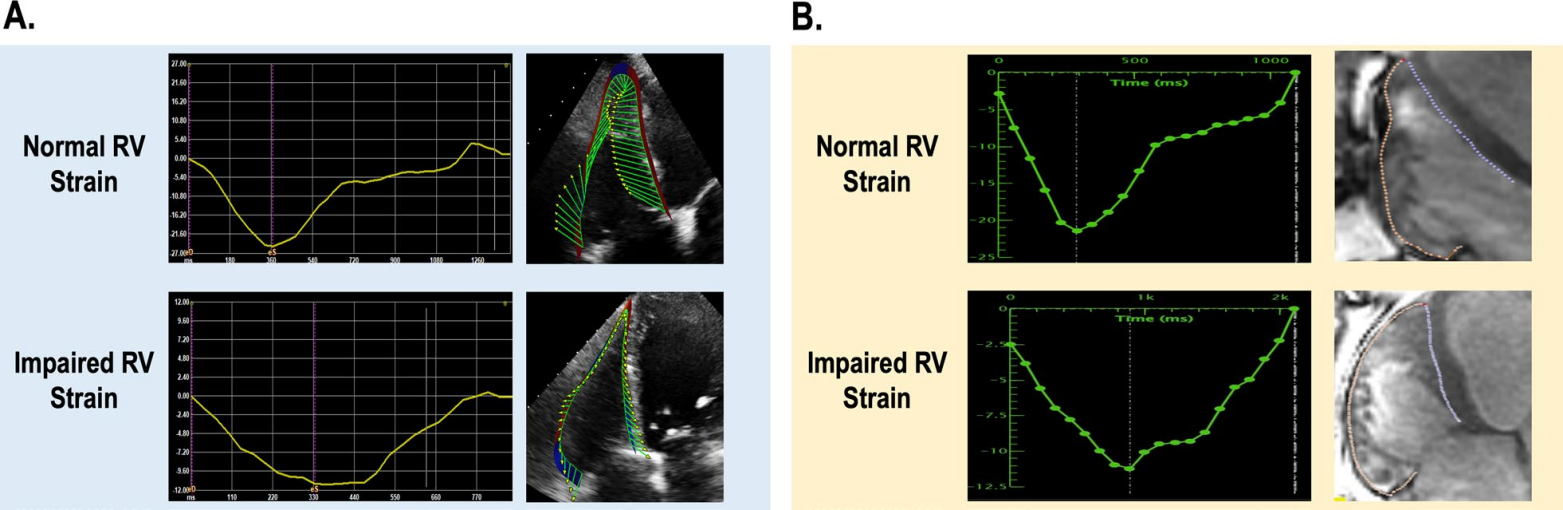
**a** Representative example of RV strain as quantified using echocardiography. Note differential peak strain characteristic of normal RV GLS (top) and impaired RV GLS (bottom). **b** Representative example of RV strain as quantified using CMR. Note differential peak strain characteristic of normal RV GLS (top) and impaired RV GLS (bottom)

STE and cine enabled FT-CMR were performed in all patients (Fig. 1). As shown in Table 2, RV GLS was lower in patients with RV_DYS_ whether quantified via STE or FT-CMR (12.5 ± 4.1% vs. 21.0 ± 3.5%; p < 0.001 and 13.7 ± 3.9% vs. 23.7 ± 5.4%; p < 0.001) which were paralleled by RV regional (septal and free wall) function with lower septal strain (STE: 7.2 ± 5.7% vs. 13.2 ± 6.7%; p < 0.001| FT-CMR: 17.2 ± 5.1% vs. 27.1 ± 6.2%; p < 0.001) and free wall strain (STE: 11.6 ± 5.5% vs. 21.6 ± 4.3%; p < 0.001 | FT-CMR: 9.0 ± 4.9% vs. 19.1 ± 5.5%; p < 0.001).

### Agreement between echo and CMR

**Fig. 2:**
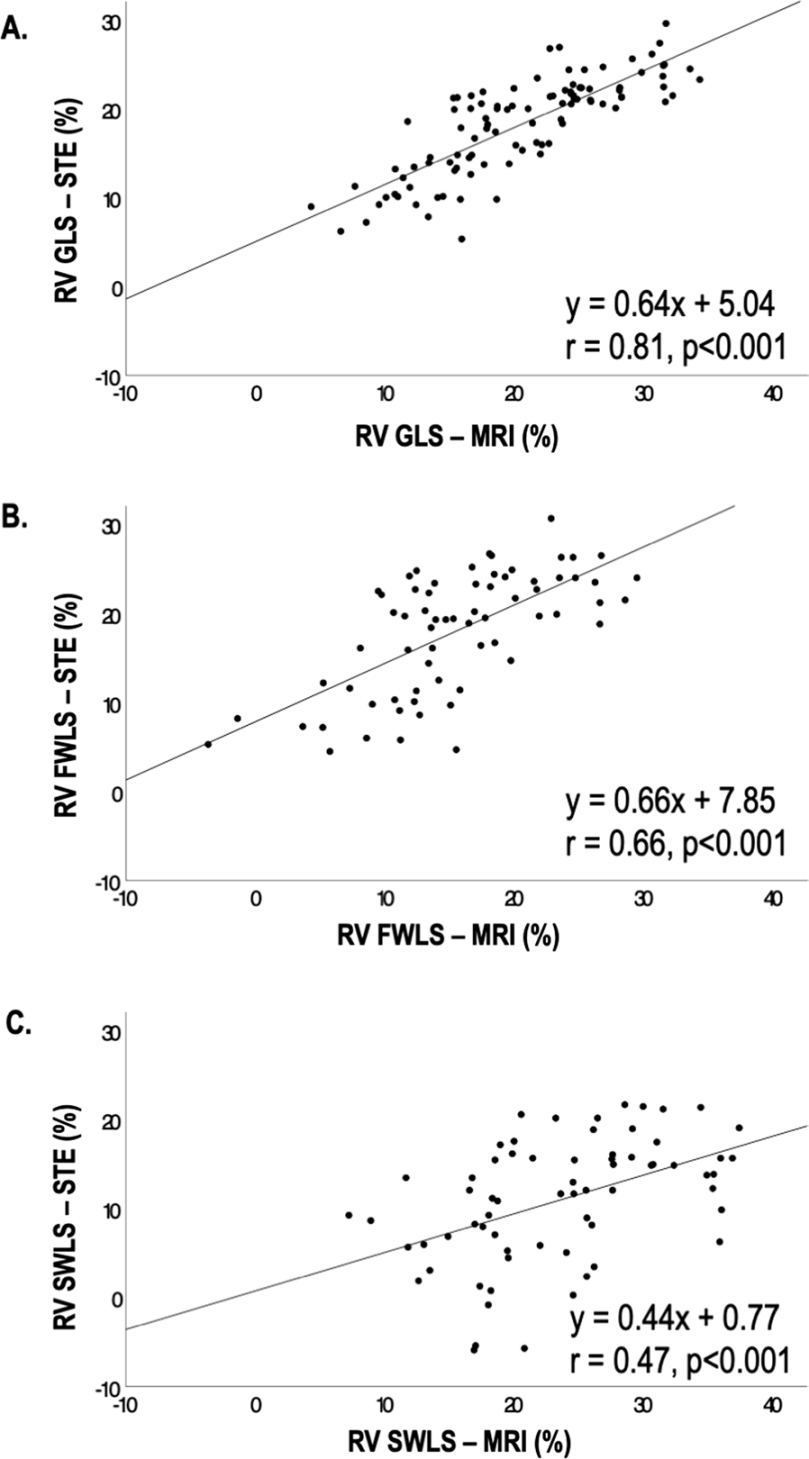
RV global (**A**), free wall (**B**), and septal (**C**) wall longitudinal strain as quantified by STE against corresponding strain as quantified by FT-CMR. As shown, STE and FT-CMR RV GLS yielded strong correlations, followed by free wall and septal strain

**Fig. 3:**
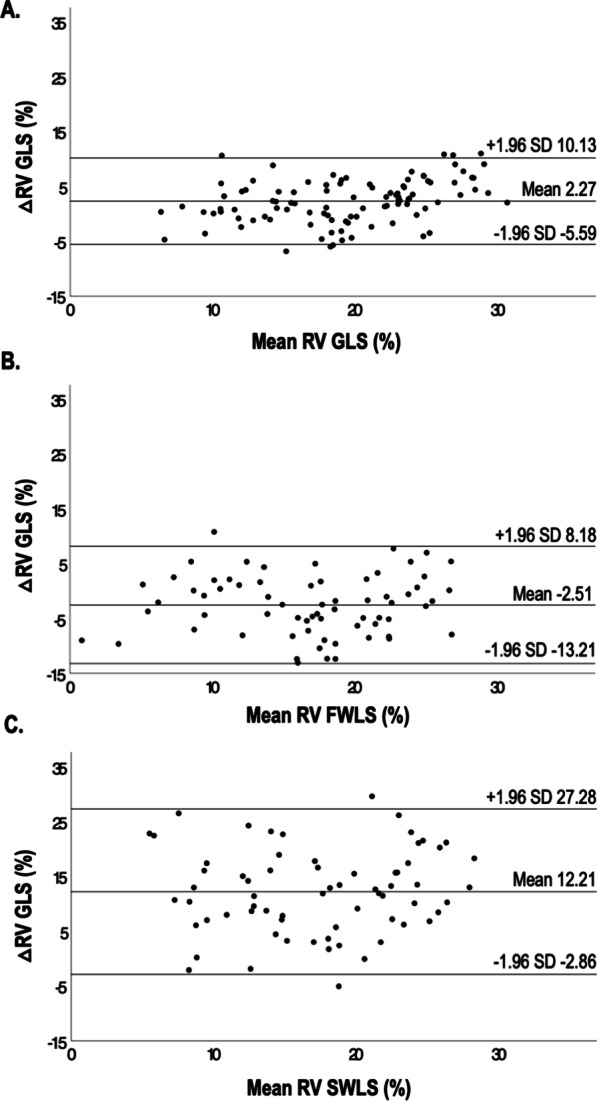
Bland–Altman plots of RV global (**A**), free wall (**B**), and septal (**C**) longitudinal RV strains by STE and FT-CMR. Of note, RV GLS was slightly underestimated by STE compared with FT-CMR. The narrowest limits of agreement were seen for RV GLS, followed by free wall and septal strain

As shown in Fig. 2, STE and FT-CMR RV GLS yielded strong correlations (r = 0.81, p < 0.001), whereas free wall strain demonstrated slightly lesser correlations (r = 0.66, p < 0.001) with weaker correlations observed for septal strain (r = 0.47, p < 0.001). Figure 3 shows Bland–Altman plots which demonstrates small underestimation of RV GLS by STE compared with FT-CMR (bias: 2.27%, 95% CI: 2.27 ± 7.86%). Narrowest limits of agreement were seen for RV GLS (LOA: ± 7.86%) and wider limits for free wall (LOA: ± 10.69%) and septal (LOA: ± 15.07%) strain.

### Diagnostic performance for RV function

**Fig. 4:**
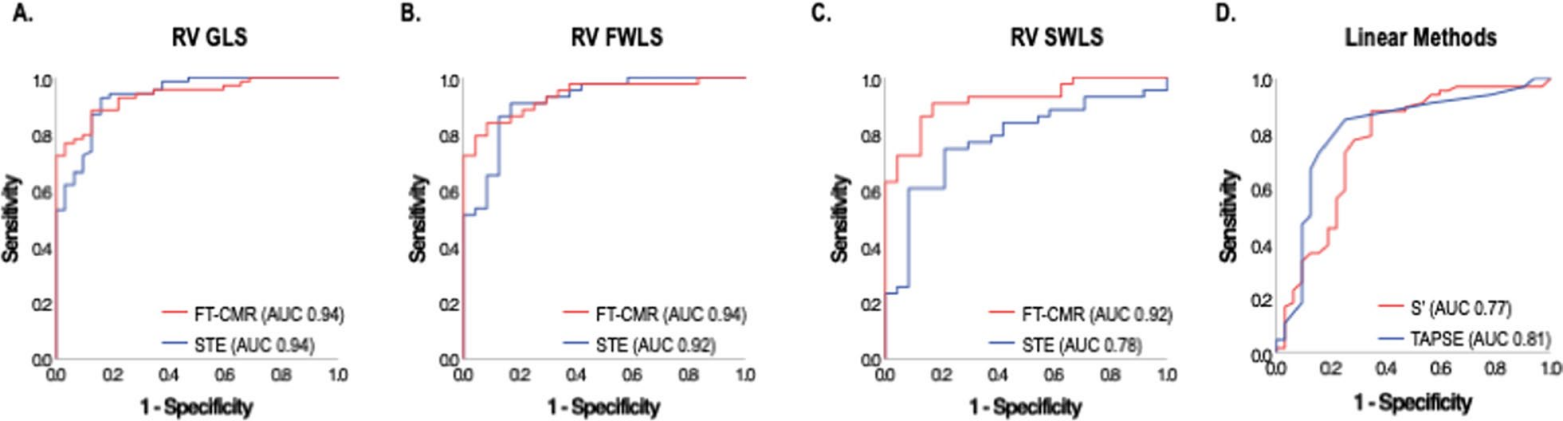
ROC analysis for the identification of RV_DYS_ using RV global (**A**), free wall (**B**), and septal (**C**) longitudinal strain as well as conventional RV indices ([**D**] TAPSE and RV S’). As shown, STE and FT-CMR had near equivalent diagnostic performance for RV GLS, followed by RV free wall and septal strain

STE and FT-CMR quantified RV strain were tested with regard to diagnostic test performance for RV_DYS_ as adjudicated by CMR defined as RVEF < 50%. As shown, ROC curves in Fig. 4 demonstrate similar excellent diagnostic performance of STE as compared to FT-CMR GLS (AUC: 0.94 for both), paralleling similar diagnostic performance when examining free wall strain for both modalities (FT-CMR AUC 0.94 and STE AUC 0.92). However, while FT-CMR demonstrated similar excellent diagnostic performance for septal wall strain (AUC 0.92), diagnostic performance of septal STE was lower (AUC 0.78). Regarding conventional linear parameters, RV S’ and TAPSE yielded overall lower diagnostic accuracy (RV S’ AUC 0.77 and TAPSE AUC 0.81).

### RV strain associated clinical outcomes

**Fig. 5:**
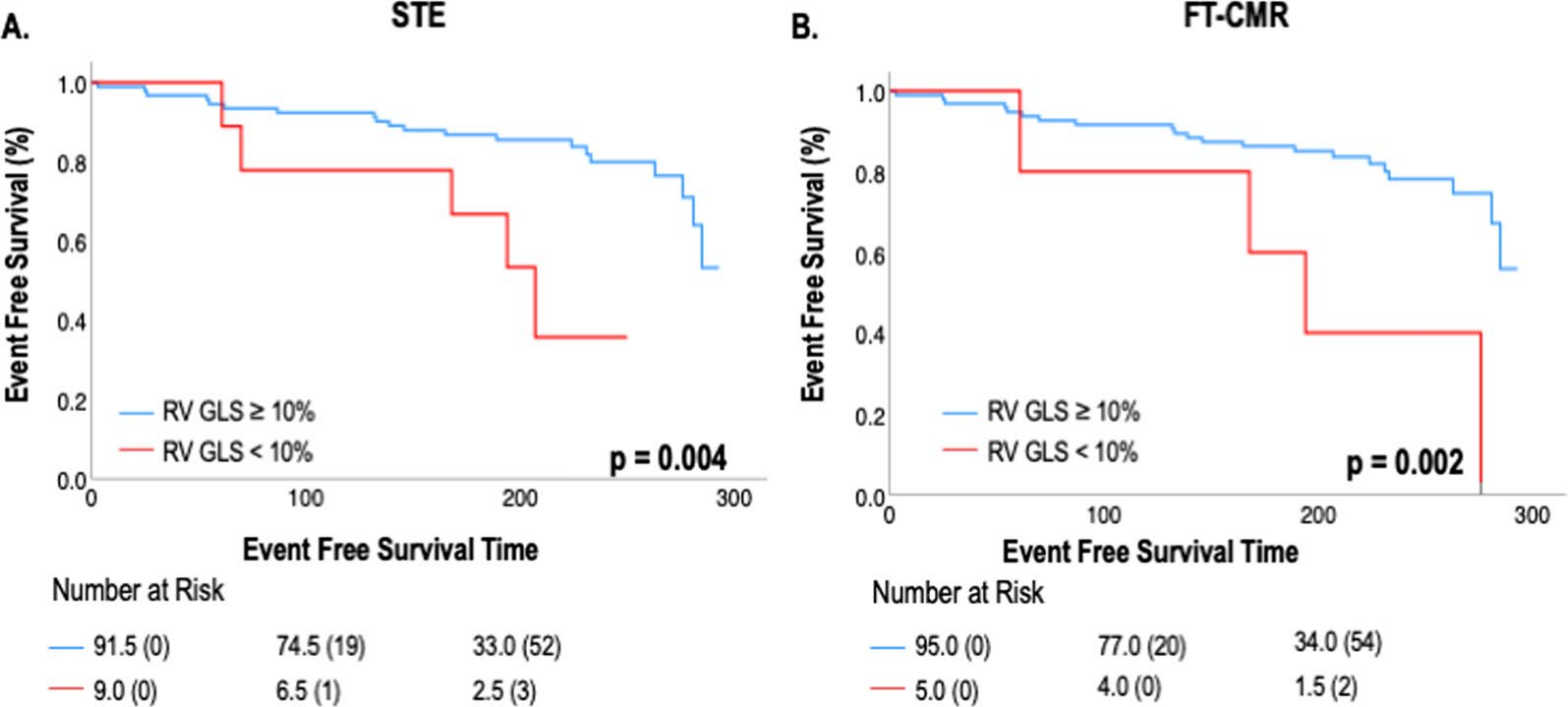
Kaplan–Meier survival analysis for patient groups stratified based on RV impairment as determined by STE (**A**) and FT-CMR (**B**) RV GLS. As shown, both STE and FT-CMR derived RV GLS impairment were associated with increased risk of all-cause mortality or HF hospitalization

During median follow up duration after imaging of 51 months (IQR 42, 60 months), 25% of the study population experienced a composite event inclusive of all-cause mortality (n = 21) and/or HF hospitalization (n = 9). Table 3a provides results for clinical and imaging indices in relation to outcome. As shown, both STE and FT-CMR derived RV GLS conferred increased risk of all-cause mortality or HF hospitalization (HR 4.06 [1.47–11.22]; p = 0.007 and HR 4.73 [1.60–14.01]; p = 0.005, respectively) whereas conventional RV parameters including RV S’ and TAPSE did not (HR 0.97 [0.84–1.12]; p = 0.69 and HR 0.57 [0.20–1.65]; p = 0.30, respectively). Of note, volumetrically derived RVEF on CMR and 3D echo were both associated with increased risk of composite outcomes (CMR RVEF HR 0.71 [0.51–0.99]; p = 0.04 and 3D RVEF HR 0.62 [0.43–0.89]; p = 0.01). Multivariable analysis (Table 3b) demonstrated abnormal RV GLS, as measured either by FT-CMR or STE, to be associated with all-cause mortality or HF hospitalization even after controlling for age (HR 5.34 [1.79–15.93]; p = 0.003 and HR 4.51 [1.62–12.58]; p = 0.004, respectively). Kaplan–Meier survival curves as stratified by RV GLS impairment by CMR and STE are shown in Fig. 5.

**Table 3:** Predictors of all-cause mortality or HF Hospitalization

	Hazard ratio (95% CI)	p
A. Univariable Cox Models for All-Cause Mortality or HF Hospitalization		
*Clinical History*		
Age (per 10 years)	1.53 (1.02–2.29)	**0.04**
Male gender	0.73 (0.29–1.84)	0.50
Hypertension	2.25 (0.67–7.54)	0.19
Diabetes mellitus	1.55 (0.69–3.46)	0.29
Family history CAD	0.91 (0.36–2.29)	0.83
Tobacco use	0.67 (0.30–1.48)	0.32
*Imaging Markers*		
***Echocardiography***		
*Right Ventricle*		
Ejection fraction (per 10%)	0.62 (0.43–0.89)	**0.01**
S' (cm/s)	0.97 (0.84–1.12)	0.69
TAPSE (cm)	0.57 (0.20–1.65)	0.30
*Left Ventricle*		
Ejection fraction (per 10%)	0.76 (0.57–1.02)	0.07
Abnormal Peak Longitudinal Strain*		
RV global (%)	4.06 (1.47–11.22)	**0.007**
RV free wall (%)	2.36 (1.01–5.52)	**0.048**
RV septal (%)	0.85 (0.35–2.10)	0.73
***CMR***		
*Right Ventricle*		
Ejection fraction (per 10%)	0.71 (0.51–0.99)	**0.04**
*Left Ventricle*		
Ejection fraction (per 10%)	0.79 (0.60–1.03)	0.08
Abnormal Peak Longitudinal Strain*		
RV global (%)	4.73 (1.60–14.01)	**0.005**
RV free wall (%)	2.36 (1.01–5.52)	**0.048**
RV septal (%)	3.36 (0.78–14.47)	0.10
B. Multivariate Cox Models for All-Cause Mortality or HF Hospitalization		
*Model 1*		χ^2^ = 13.34, p = 0.001
Age (per 10 years)	1.60 (1.06–2.41)	**0.03**
Abnormal RV GLS on STE (%)†	4.51 (1.62–12.58)	**0.004**
*Model 2*		χ^2^ = 14.43, p < 0.001
Age (per 10 years)	1.60 (1.06–2.41)	**0.03**
Abnormal RV GLS on CMR (%)†	5.34 (1.79–15.93)	**0.003**

Bold values identify a significance of *p* < 0.05

^*^Peak longitudinal strain < 10%

^†^RV GLS < 10%

## Discussion

This is the first study to date to our knowledge to directly compare RV GLS via STE and FT-CMR with respect to its relative diagnostic performance as well as prognostication for all-cause mortality and heart failure hospitalization among patients with functional mitral regurgitation. The main findings of our study are as follows: First, RV strain via STE and FT-CMR generally agreed closely with one another with strongest correlations demonstrated for RV GLS and modest correlations for septal and free wall strain. Additionally, while strain values correlated closely, there was a small underestimation of RV GLS by STE compared to FT CMR. Second, whereas STE and FT-CMR RV global strain assessment yielded nearly equivalent diagnostic performance in relation to the reference standard of CMR defined RV_DYS_, diagnostic performance of septal STE for discriminating global RV_DYS_ was poor. Finally, impaired RV GLS stratified adverse prognosis with similar incremental predictive value yielded by CMR and echo over conventional RV indices (RV S’ and TAPSE). Taken together, our findings support the concept that RV GLS via STE and FT-CMR are robust markers of RV_DYS_ with each providing incremental prognostic value, though importantly, absolute GLS parameters acquired from each modality are not interchangeable.

Our findings are supported by and extend on prior research with respect to RV strain quantification across modalities. Recent work by Taha et al. showed moderate correlations between RV strain values obtained by STE and FT-CMR in 110 patients with arrhythmogenic right ventricular cardiomyopathy [17]. In this study, RV free wall longitudinal strain (FWLS) showed near equivalent correlations between the two modalities (r = 0.58, p < 0.05) though septal and average strains were not specifically examined. Similarly, Erley et al. studied 62 patients undergoing both CMR and echo and demonstrated moderate intramodality agreement between STE and FT-CMR for free wall (r = 0.62, p < 0.001) and global strain (r = 0.54, p < 0.001) [18]. While promising, this latter study was limited by its retrospective nature and neither study examined the relative prognostic value of GLS by modality.

Regarding differences in absolute value of GLS by STE and FT-CMR, there are several factors at play which contribute to non-uniformity in strain assessment across modalities. First, there are modality dependent differences in how the RV is imaged leading to differences in scanning angle on echo and CMR. The complicated contour and position of the RV is particularly susceptible to small changes in scanning angles which have the potential to lead to differences in reproducibility and accuracy. Secondly, there are differences in resolution between the two modalities with feature tracking strain having higher signal to noise ratio and spatial resolution whereas STE generally has higher temporal resolution. It should also be noted that STE tracking may be difficult in patients with suboptimal endocardial definition and thus GLS calculations may be superior via FT-CMR in these cases. Additionally, regarding differential diagnostic and prognostic performance of free wall and global RV strain, we note that the interventricular septum is an integral component of LV function and therefore, it is possible that better prognostic performance of RV global strain vs. free wall strain observed in this study may reflect composite LV and RV assessment for RV global strain.

RV GLS is an emerging marker of RV_DYS_ which provides incremental diagnostic and prognostic value as compared to standard assessment in patients across a wide spectrum of conditions including HF and CAD [11, 19]. Regarding strain, given that the RV is primarily composed of longitudinally aligned myofibers (rather than circumferential fibers in the subepicardium), longitudinal right ventricular strain (RV GLS) best represents RV performance [20]. Early detection of RV_DYS_ in mitral regurgitation is particularly important as prompt diagnosis has the potential to impact timing and nature of intervention. RV_DYS_ as assessed via conventional parameters has been shown to be an adverse prognostic marker among patients with advanced mitral regurgitation [21, 22] and has important long-term prognostic implications among patients undergoing mitral valve intervention [22, 23]. Earlier detection of RV_DYS_ via GLS may provide additional risk stratification for mitral regurgitation to identify patients who may benefit from more prompt intervention.

Additionally, even among patients with CAD alone, RV_DYS_ is known to confer serious clinical consequences. Mortality risk is increased up to threefold in patients with RV_DYS_, among whom prognosis varies in proportion to magnitude of RV contractile impairment [9, 24]. RV_DYS_ also impacts morbidity: for example, RV dysfunction increases the risk of clinical heart failure and hospital admissions [25]. Effort capacity is also impacted by the RV. In patients with known or suspected CAD, our prior research has shown RV_DYS_ to independently impair exercise tolerance [26]. Despite advances in therapeutic approaches for CAD, management approaches for RV_DYS_ remain limited. One reason for uncertainty regarding RV_DYS_ in patients with CAD stems from limitations in imaging methods that have been used for RV assessment. RV GLS has the potential to overcome limitations of conventional assessment and provide incremental value to detection of RV dysfunction among patients with CAD.” Despite advances in therapeutic approaches for CAD, management approaches for RV dysfunction remains limited. One reason for uncertainty regarding RV dysfunction in patients with CAD stems from limitations in imaging methods that have been used for RV assessment. RV GLS has the potential to overcome limitations of conventional assessment and provide incremental value to detection of RV dysfunction among patients with CAD. Our study examining RV strain across modalities with respect to both its diagnostic and prognostic performance provides additional evidence of the robustness of this technique.

Several limitations should be noted in this study. First, this study examined RV performance in patients with functional mitral regurgitation and thus further validation studies are needed to extrapolate these findings to broader cohorts. Second, our CMR and echo exams were performed for research purposes and thus may have yielded higher diagnostic and prognostic performance as compared to that derived from routine clinical exams. On the other hand, this study was designed to test characteristics of multiparametric RV functional indices acquired under optimal conditions and identify whether systematic offsets exist between strain and RV indices, so as to inform larger scale applications in both clinical and epidemiologic research applications. Lastly, it is important to keep in mind that given uncertainty regarding optimal cutoff and wide variation in reported RV GLS threshold for prediction of outcomes [11, 27–30], our study used a singular cutoff (< 10%). However, it should also be recognized that our findings of an association of GLS with outcomes was paralleled by other volumetric RV parameters including RVEF on echo and CMR. In this context, this study adds to the growing body of literature that demonstrate RV impairment as an important prognostic marker among patients with mitral regurgitation.

## Conclusions

This study demonstrates RV GLS as quantified by both CMR and echo to yield close agreement and near equivalent diagnostic performance for volumetric RV_DYS_ and incremental prognostic utility among patients with functional mitral regurgitation. Further studies are warranted to test whether strain-based assessments of RV_DYS_ provide utility for informing therapeutic decision making in patients with mitral regurgitation and whether longitudinal changes in strain parallel differential clinical prognosis.

## Data Availability

The datasets used and/or analyzed during the current study are available from the corresponding (JK) author on reasonable request.

## Abbreviations

AUC: Area under the curve
CAD: Coronary artery disease
CMR: Cardiac magnetic resonance
EF: Ejection fraction
FT-CMR: Feature tracking cardiac magnetic resonance
HF: Heart failure
LOA: Limits of agreement
ROC: Receiver operating characteristic
RV_DYS_: Right Ventricular dysfunction
GLS: Global longitudinal strain
STE: Speckle tracking echocardiography
